# Phase Selection Method for 10 kV Three-Core Cables Under Single-Phase Grounding Fault Transient Based on Surface Magnetic Field Sensing

**DOI:** 10.3390/s26031016

**Published:** 2026-02-04

**Authors:** Hang Wang, Tianhu Weng, Wenfang Ding, Shuai Yang, Zheng Xiao, Hang Li, Jun Chen

**Affiliations:** 1 Hubei Engineering Research Center for Safety Monitoring of New Energy and Power Grid Equipment, Hubei University of Technology, Wuhan 430068, China; wanghang@whu.edu.cn (H.W.); w19980924w@163.com (T.W.); wenfangding@163.com (W.D.); ys3254@163.com (S.Y.); 2 State Grid Hubei Electric Power Company, State Grid Corporation of China, No. 1701, Jiefang Road, Jiangan District, Wuhan 430013, China; cissy0080@126.com (Z.X.); leehang@outlook.com (H.L.)

**Keywords:** fault phase selection, surface magnetic field sensing, single-phase grounding, transient three-core cable

## Abstract

Single-phase grounding is the dominant fault type in urban power distribution networks. Because the total magnetic flux would not change around the cable under a single-phase grounding fault, ferromagnetic zero-sequence current sensors cannot distinguish the faulted phase of belted cables, which are the main type in 10 kV distribution networks. To fill this gap, a two-step methodology is proposed using an annular TMR magnetic sensor to measure the magnetic field intensity at six points on the cable surface and to distinguish the faulted phase using the magnetic field intensity differences between the TMRs. The first step is calculating the rotation angles between the six magnetic sensors and the three cable cores after installation. A differential evolution algorithm is used to calculate the rotation angles in the sensing model. The second step is to detect the fault phase under a single-phase grounding fault transient, with the magnetic field intensity difference taken as the criterion. The methodology is verified through simulation and experiment. The results show that the relative errors of the rotation angles are all less than 1%. Under a single-phase grounding fault, the faulted phase can be accurately identified. The proposed method can effectively identify the faulted phase of 10 kV three-core cables under single-phase grounding and has significant engineering application value.

## 1. Introduction

Cables are the backbone of the modern power distribution grid. For instance, the total length of medium-voltage (MV) cable circuits in China had exceeded one million kilometers by the end of 2025, with an average annual growth rate of 7% over the past decade [1,2]. In medium-voltage distribution networks, single-phase grounding faults are the most common type of fault, accounting for more than 70% of the total. Medium-voltage cable-based distribution networks utilize three-core cables as power lines; these cables feature a belted structure and offer high safety performance as well as strong electromagnetic interference suppression capability [3]. For this reason, the centralized laying of multi-circuit three-core cables is quite prevalent in areas with dense power loads. However, underground cables are susceptible to multiple factors, including third-party construction damage, mechanical stress, and environmental temperature and humidity fluctuations. These factors can degrade the insulation performance of cables and consequently lead to single-phase grounding faults [4]. Accurate identification of the fault phase is crucial for the rapid restoration of the power supply. Nevertheless, traditional methods require invasive operations at both ends of the cable, which increases the maintenance workload and prolongs the fault handling cycle. Therefore, there is an urgent need to develop more efficient and intelligent fault phase identification methods to enhance the overall reliability of power supply systems.

In previous work, phase selection methods in MV power distribution grid mainly focused on overhead lines rather than cables. Due to differences in line structures and parameters [5], overhead lines and three-core cable lines exhibit distinct voltage and current characteristics after single-phase grounding faults. Overhead lines feature small ground capacitance, pronounced zero-sequence voltage and current characteristics, and rich transient components, enabling effective fault identification based on voltage and current features [6]. In contrast, three-core cable lines exhibit large ground capacitance and strong inter-phase coupling, resulting in capacitive-dominated zero-sequence currents [7], weak phase discrimination, and rapidly decaying transients, which significantly complicate faulty phase identification. Aiming at this key problem of fault phase selection in distribution networks, scholars at home and abroad have carried out systematic and in-depth research and proposed a variety of fault phase selection methods with different characteristics. In the field of fault phase selection based on abrupt quantities, References [8,9] proposed a phase selection method based on power–frequency amplitude differences in phase-to-phase current fault components. This method has been widely applied in transmission networks, but its application effectiveness is limited due to the complexity of distribution networks. In terms of steady-state phase selection research, Reference [10] uses a single sequence component to reflect all fault types and comprehensively identify fault phases; however, it requires a long data acquisition window, and when the system operation mode changes, the characteristics may weaken or even disappear, resulting in poor phase selection performance. In research on transient phase selection, Reference [11] takes the transient current of short circuit faults as the analysis object and proposes a fault phase selection method based on wavelet entropy weight. In addition, Reference [12] extracts the modulus phasor of a single frequency of fault current using the S-transform and puts forward a phase selection method based on modulus relationships and phase current integration. Although these methods have high phase selection accuracy, they are susceptible to the influence of system parameters, and their effectiveness has not been verified in engineering practice. Reference [13] introduces deep learning into this field and realizes automatic phase selection with strong anti-interference ability through convolutional neural networks; however, its engineering application is restricted by problems such as data acquisition and model training.

The present authors have invented a Hall-element-based surface magnetic field sensor and used it for phase current measurement; however, it was not used in transient magnetic sensing or phase selection [14,15]. In recent years, with the rapid development of magnetic field sensing technology, using magnetic fields instead of electric currents as characteristic quantities in distribution systems has emerged as a new research trend [16]. Traditional distribution network monitoring primarily relies on invasive current sensors, which require circuit interruption for installation and thus increase the difficulty of system maintenance. In contrast, TMR magnetic sensors have demonstrated broad application prospects in distribution network monitoring due to their advantages, such as high sensitivity, strong anti-interference capability, and non-invasiveness. Particularly in monitoring applications of three-core cables, given that such cables adopt an integral sheath structure, sensors can only be arranged outside the cables, which imposes higher requirements on sensor arrangement and detection accuracy. To address this challenge, relevant studies have proposed various solutions. For instance, Reference [17] proposes a novel magnetic field sensing technique for detecting underground power cables and verifies its effectiveness using simulation models of 11 kV and 132 kV underground power cables. Reference [18] presents a novel coreless current sensor unit for measuring electrical current in circular conductors. Unlike conventional designs, these sensors do not need to be fixed directly onto the conductor, which significantly enhances their flexibility and ease of deployment. Reference [19] proposes a theoretical and experimental method for evaluating crosstalk interference in a circular array of fluxgate sensors used for current measurement. Reference [20] investigates a differential array of magnetic field sensors. In this differential approach, the Moore–Penrose inversion method is used to obtain the set of currents at different positions inside the sensor by measuring the radial difference in the magnetic fields. Reference [21] provides an in-depth analysis of measurement errors in circular magnetic sensor arrays used for current measurement. By introducing a theoretical framework based on vector mathematics, the paper investigates the effects of conductor position variations on measurement accuracy. Reference [22] designed an annular magnetic field sensor array with a 45° mutual spacing, which effectively expanded the angular coverage of magnetic field detection. References [23,24] developed an annular giant magnetoresistive sensor array with a 90° mutual spacing tailored to the monitoring requirements of three-core cables; by increasing the sensor arrangement density, the accuracy of magnetic field measurement was significantly improved. Reference [18] constructed a sensor array composed of three Hall sensors with a 120° mutual spacing and further enhanced the detection capability for superimposed magnetic fields in cables by optimizing the spatial layout between sensors. Reference [14] designed an annular Hall sensor array with a 60° mutual spacing, whose research mainly focused on the inversion of steady-state phase currents. Reference [15] considered the influence of the steel tape shield layer of three-core cables on the inverted phase current, but neither involved engineering application research in the field of distribution network fault diagnosis. Despite the remarkable progress made in existing research on surface magnetic field sensing for three-core cables [25], its practical application in the field of single-phase grounding fault diagnosis remains relatively limited, and there is still a lack of effective methods for single-phase grounding fault phase selection.

In this paper, an annular TMR magnetic sensor is adopted to realize non-invasive fault phase selection for single-phase grounding faults in three-core cables. First, a differential evolution algorithm is used to invert the rotation angle under steady-state system conditions. Then, the difference value is selected as the time-domain characteristic quantity, and six measurement points are arranged on the surface of the annular TMR magnetic sensor as spatial characteristic quantities; the magnetic field intensity difference is used to distinguish the fault phase at the moment of a single-phase grounding fault transient. The feasibility of the above-mentioned phase selection method is analyzed via simulation, and it is verified that this method is not affected by factors such as rotation angle or neutral grounding mode. Finally, systematic verification of the proposed phase selection method based on the annular TMR magnetic sensor is carried out on a 10 kV cable network prototype test platform. Test results demonstrate that this method effectively selects the fault phase for single-phase grounding faults in three-core cables, offering a novel approach to developing fault phase selection technology in distribution networks.

## 2. Materials and Methods

### 2.1. Single-Phase Grounding Induced Sudden Variation in Surface Magnetic Field in Three-Core Cables

#### 2.1.1. Theoretical Analysis of Surface Magnetic Field in Single-Phase Grounding of Three-Core Cables

**Figure 1:**
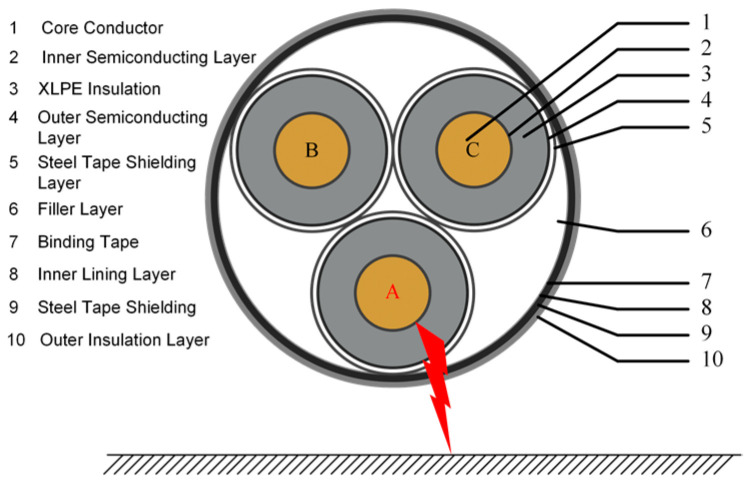
Single-phase grounding fault of a three-core cable.

The main causes of single-phase grounding faults in three-core cables are external force damage and insulation breakdown. As shown in Figure 1, a grounding loop is formed between the single-phase conductor and the earth.

**Figure 2:**
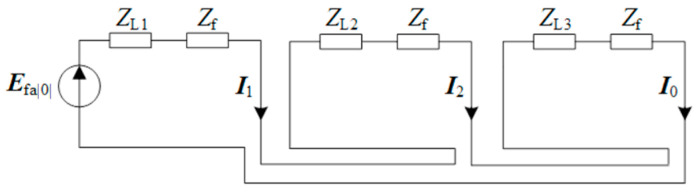
Sequence network diagram.

When a single-phase grounding fault occurs in a distribution network cable, the three-phase currents exhibit unbalanced characteristics. The symmetric component method yields Equation (1), and the composite sequence network diagram of single-phase grounding is shown in Figure 2. Here, *Z*_L1_, *Z*_L2_, and *Z*_L3_ are the positive-, negative-, and zero-sequence equivalent impedances of the line, *Z*_f_ is the fault point impedance, and ***E***_fa|0|_ is the equivalent voltage source.

(1) $$\left\{\begin{array}{l} I_{A}=I_{1}+I_{2}+I_{0}\\ I_{B}=a^{2}I_{1}+aI_{2}+I_{0}\\ I_{C}=aI_{1}+a^{2}I_{2}+I_{0} \end{array}\right.$$

where ***I***_A_, ***I***_B_, and ***I***_C_ are the A-phase, B-phase, and C-phase currents, respectively; ***I***_1_, ***I***_2_, and ***I***_0_ are the positive-sequence, negative-sequence, and zero-sequence currents, respectively; and a is the rotation factor.

During a single-phase grounding fault, the voltage of the fault phase drops, and the phase current increases, while the current of the healthy phases changes slightly. The fault phase current is expressed as Equation (2):

(2) $$I_{fa}=I_{1}+I_{2}+I_{3}=\frac{3E_{fa[0]}}{Z_{L1}+Z_{L2}+Z_{L3}+3Z_{f}}$$

The surface magnetic field distribution of three-core cables can be calculated using the Biot–Savart Law, whose integral form is shown in Equation (3):

(3) $$B=\int_{L}\frac{\mu_{0}}{4\pi }\frac{Idl\times e_{r}}{r^{2}}$$

where ***B*** is the magnetic field intensity vector, *L* is the closed integral path, *I* is the source current, *d**l*** is the current element, ***e****_r_* is the unit vector from the current element to the field point to be solved, *r* is the distance from the current element to the field point to be solved, and *μ_0_* is the vacuum permeability.

Based on the law of surface magnetic field distribution of three-core cables, a magnetic field vector superposition model of three-core cables is established. The synthetic magnetic field generated by the three-phase conductors can be expressed as Equation (4):

(4) $$B=B_{A}(r_{A},\theta_{A})+B_{B}(r_{B},\theta_{B})+B_{C}(r_{C},\theta_{C})$$

where ***B***_A_(*r*_A_, *θ*_A_), ***B***_B_(*r*_B_, *θ*_B_), and ***B***_C_(*r*_C_, *θ*_C_) are the magnetic field components generated by the A-, B-, and C-phase conductors at the measurement points (*r*_A_, *θ*_A_), (*r*_B_, *θ*_B_), and (*r*_C_, *θ*_C_), respectively.

**Figure 3:**
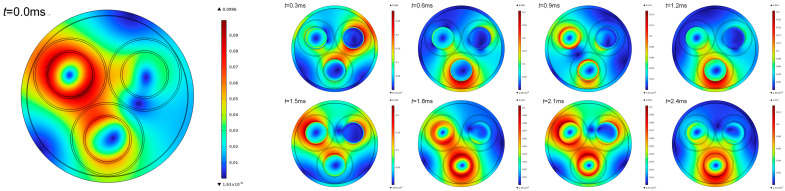
Magnetic field intensity caused by single-phase grounding in a three-core cable.

Under steady-state equilibrium conditions, the three-phase currents are 120° out of phase with each other, and the synthetic magnetic field on the surface of the three-core cable presents a uniform distribution. The magnetic field intensity changes along an elliptical trajectory with time, showing the characteristics of a rotating magnetic field [14]. When a single-phase grounding fault occurs, the fault phase current mutates, and the system changes from three-phase operation to two-phase operation, resulting in distortion of the synthetic magnetic field, as shown in Figure 3. It is assumed that the system is in steady-state operation at 0 ms, a single-phase grounding fault occurs at 0.3 ms, and the synthetic magnetic field on the surface of the three-core cable increases significantly during the single-phase grounding fault transient stage from 0.3 ms to 2.4 ms.

Assuming an A-phase single-phase grounding fault occurs, the fault current is shown in Equation (5), and the magnetic field component of the fault phase A is shown in Equation (6). Here, *I*_f_ is the amplitude of the fault current, *ψ*_f_ is the phase angle of the fault current, and *d* is the distance from the conductor center to the cable center.

(5) $$I_{A}=I_{m}\cos(\omega t)+I_{f}\cos(\omega t+\psi_{f})$$

(6) $$\Delta B_{r}(r,0)=\frac{\mu_{0}I_{f}}{2\pi }\cdot \frac{d_{A}\sin(\psi_{f})}{(r^{2}+d_{A}^{2}-2rd_{A}{)}^{3/2}}$$

The results show that when a single-phase grounding fault occurs in a three-core cable, the surface magnetic field near the fault phase changes most significantly. The fault phase can be identified by measuring the magnetic field changes at multiple positions on the surface of the three-core cable.

#### 2.1.2. Annular TMR Magnetic Array Model and Phase Selection Characteristic Quantities

**Figure 4:**
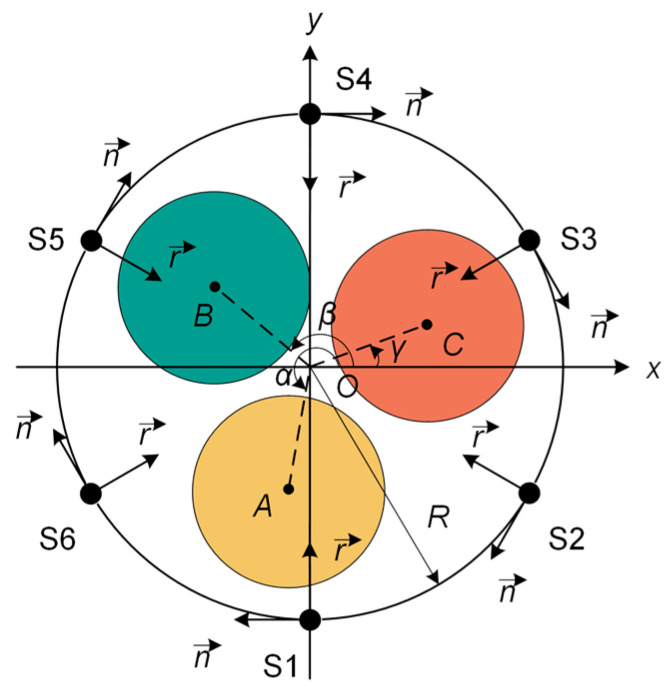
Surface magnetic field sensing model of a three-core cable.

This paper refers to six TMR sensors with a mutual spacing of π/3 from Reference [14], numbered S1~S6, which are placed at any position on the surface of the three-core cable without being installed on the extension line connecting the cable center and the phase center, as shown in Figure 4. Point *O* is the center of the three-core cable, points *A*, *B*, and *C* are the centers of the A-, B-, and C-phase cores, respectively. The distances from point *O* to the centers of each core are *r*_A_, *r*_B_, and *r*_C_, respectively, and the radius of the cable is *R*. The rotation angles between *OA*, *OB*, *OC* and the positive direction of the *x*-axis are *α*, *β*, and *γ*, respectively, with a mutual difference of 2π/3. From the trigonometric relationship, the coordinates of points *A*, *B*, and *C* are (*r*_A_*cosα*, *r*_A_*sinα*), (*r*_B_*cosβ*, *r*_B_*sinβ*), and (*r*_C_*cosγ*, *r*_C_*sinγ*), respectively. The tangential magnetic field intensities *B*_S1_~*B*_S6_ along *n* are generated at S1~S6. The magnitudes of the magnetic field intensities at the six measurement points are related to the three-phase currents *I*_A_, *I*_B_, and *I*_C_ and the coefficients before the phase currents. Among these, the spatial distances between points *A*, *B*, *C* and each measurement point are the key factors determining the coefficients. The influence of the coefficients will make the six measurement points obtain different magnetic field intensities at the same time.

The six measurement points *B*_S1_*~B*_S6_ arranged on the surface of the annular TMR array are composed of two groups of surface measurement points, namely *B*_S1_, *B*_S3_, *B*_S5,_ and *B*_S2_, *B*_S4_, *B*_S6_. Adding the surface magnetic fields of the three measurement points *B*_S1_, *B*_S3_, and *B*_S5_ gives the *B*_M_ expression as shown in Equation (7), and adding the surface magnetic fields of the three measurement points *B*_S2_, *B*_S4_, and *B*_S6_ gives the *B*_N_ expression shown in Equation (8):

(7) $$B_{M}=\frac{\mu_{0}}{2\pi }\left(I_{A}+I_{B}+I_{C}\right)*\sum_{k=1}^{3}\frac{R-r\sin(\alpha +\theta_{k}^{\left(1\right)})}{R^{2}+r^{2}-2Rr\sin(\alpha +\theta_{k}^{\left(1\right)})}$$

where $\theta_{1}^{\left(1\right)}$ = −*π*, $\theta_{2}^{\left(1\right)}$ = *π/3*, and $\theta_{3}^{\left(1\right)}$ = −*π/3*.

(8) $$B_{N}=\frac{\mu_{0}}{2\pi }\left(I_{A}+I_{B}+I_{C}\right)*\sum_{k=1}^{3}\frac{R-r\sin(\alpha +\theta_{k}^{\left(2\right)})}{R^{2}+r^{2}-2Rr\sin(\alpha +\theta_{k}^{\left(2\right)})}$$

where $\theta_{1}^{\left(2\right)}$ = 2π/3, $\theta_{2}^{\left(2\right)}$ = 0, and $\theta_{3}^{\left(2\right)}$ = −2π/3.

Analysis of Equations (7) and (8) shows that both *B*_M_ and *B*_N_ are related to the zero-sequence current. In a three-phase system, the zero-sequence current expression is:

(9) $$I_{0}=\frac{I_{A}+I_{B}+I_{C}}{3}$$

where *I*_0_ is the zero-sequence current of the three-phase system.

Introducing the zero-sequence current into *B*_M_ and *B*_N_, Equations (7) and (8) can be simplified as follows:

(10) $$B_{M}=\frac{3\mu_{0}}{2\pi }I_{0}\cdot \sum_{k=1}^{3}\frac{R-r\sin(\alpha +\theta_{k}^{\left(1\right)})}{R^{2}+r^{2}-2Rr\sin(\alpha +\theta_{k}^{\left(1\right)})}$$

(11) $$B_{N}=\frac{3\mu_{0}}{2\pi }\;I_{0}\cdot \sum_{k=1}^{3}\frac{R-r\sin(\alpha +\theta_{k}^{\left(2\right)})}{R^{2}+r^{2}-2Rr\sin(\alpha +\theta_{k}^{\left(2\right)})}$$

Adding *B*_M_ and *B*_N_ gives the superimposed magnetic field of the six measurement points as follows:

(12) $$B_{SUM}=\frac{3\mu_{0}}{2\pi }\;I_{0}\;\sum_{k=1}^{6}\frac{R-r\sin(\alpha +\theta_{k})}{R^{2}+r^{2}-2Rr\sin(\alpha +\theta_{k})}$$

where *θ*_1_ = −*π*, *θ*_2_ = 2*π*/3, *θ*_3_ = *π*/3, *θ*_4_ = 0, *θ*_5_ = −*π*/3, and *θ*_6_ = −2*π*/3.

The above analysis shows that the superimposed magnetic field *B*_SUM_ of the six measurement points *B*_S1_~*B*_S6_ is positively correlated with the zero-sequence current and has nothing to do with the positive-sequence current and negative-sequence current. In a three-phase balanced system, the zero-sequence current is zero; therefore, the superimposed magnetic field *B*_SUM_ is also zero. When an asymmetric fault occurs in the system, such as a single-phase grounding fault in a three-core cable, the zero-sequence current is non-zero, which in turn generates a superimposed magnetic field *B*_SUM_, leading to changes in the surface magnetic field of the six measurement points of the annular TMR array.

### 2.2. Phase Selection Method for Single-Phase Grounding Faults in Three-Core Cables

#### 2.2.1. Solution of the Rotation Angle of the Steady-State TMR Array

Under steady-state system conditions, the rotation angles are inverted using the surface magnetic field formulas of the six measurement points *B*_S1_~*B*_S6_, and the planar relative arrangement of the annular TMR array and the A-, B-, and C-phase conductors is determined according to *α*, *β*, and γ. Among them, the rotation angles *α*, *β*, and *γ* are unknown quantities, whereas the cable radius *R* and the distances *r*_A_, *r*_B_, and *r*_C_ from the cable center to the three-phase core centers are known quantities. The oscilloscope detects the three-phase currents *I*_A_, *I*_B_, and *I*_C_, and the TMR sensor acquires the surface magnetic field intensity *B*_S1_~*B*_S6_ of the three-core cable. The equations contain sine terms related to the rotation angles and are nonlinear. There are three unknowns, *α*, *β*, and *γ* in the equations, and the number of unknowns is less than the number of equations. Solving the equations can yield numerical solutions.

Converting a system of nonlinear equations into an optimization problem is a widely adopted solution approach [26,27,28], and the key to this method lies in constructing an appropriate fitness function. Based on the differential evolution algorithm [29], to solve for the rotation angles *α*, *β*, *γ* during the steady-state operation of the system, it is necessary to first construct the fitness function *F* by using the inversion-calculated values and actual measured values of the surface magnetic fields *B*_S1_~*B*_S6_ at six measurement points, as shown in Equation (13):

(13) $$F=\sum_{i=1}^{6}(B_{Si}-B_{{Si}^{*}}{)}^{2}$$

where *B*_Si_ is the theoretically calculated value of the magnetic field at the measurement point, and *B*_Si*_ is the sensor-measured value at the corresponding measurement point. The values of *α*, *β*, and *γ* when the fitness function *F* takes the minimum value are the optimal solutions to the equations.

The optimization problem satisfies the following constraint conditions of the fitness function:

(14) 0≤α,β,γ≤2π

where *α*, *β*, and *γ* are the rotation angles to be solved, and their ranges are from 0 to 2π.

#### 2.2.2. Fault Phase Selection for Single-Phase Grounding Fault Transient of Three-Core Cables

The essence of judging the fault phase using the annular TMR array under transient conditions is to find the mutual relationship between the surface magnetic field intensity of the six measurement points with a mutual spacing of π/3 and the fault and healthy phases. For this reason, the concept of the difference value at each measurement point is proposed, as shown in Equation (15):

(15) $$B_{{Si}^{pp}}=B_{{Si}^{max}}-B_{{Si}^{min}}$$

where i = 1, 2, …, 6; *B*_Si^pp^_ is the transient magnetic field intensity difference at the measurement point, *B*_Si^max^_ is the maximum transient magnetic field intensity at the measurement point, and *B*_Si^min^_ is the minimum transient magnetic field intensity at the measurement point.

The surface magnetic field intensity of three-core cables has the physical characteristic of decaying with distance. The spatial distance between the six measurement points and the fault phase conductor is correlated with the measured magnetic field intensity. When a single-phase grounding fault occurs in a three-core cable, among the six measurement points of the sensor surrounding the three-core cable, the measurement point closest to the fault phase will obtain the maximum magnetic field intensity difference.

Based on this, the criterion for identifying the fault phase is established: at the moment of the single-phase grounding fault transient, among the six measurement points of the annular TMR magnetic sensor, the phase pointed to by the measurement point with the maximum magnetic field intensity difference along the radial direction r is the fault phase.

##### Time-Domain Characteristic Quantities

To evaluate the optimal time-domain characteristic quantities of the surface magnetic field transient response during single-phase grounding faults in three-core cables, a comparative analysis of the maximum and minimum values, rate-of-change, and the proposed difference value is carried out, as shown in Table 1. By simulating the change process of the transition resistance from low-resistance to high-resistance under random noise interference, phase selection accuracy is used as the evaluation criterion to quantify the performance of each characteristic quantity in terms of fault phase selection reliability.

The maximum and minimum values of the surface magnetic field at each measurement point are shown in Equation (16):

(16) $$B_{{Si}^{M}}=\left(\left|B_{{Si}^{max}}|,|B_{{Si}^{min}}\right|\right)$$

The rate-of-change in the surface magnetic field at each measurement point is shown in Equation (17):

(17) $$\frac{dB_{Si}}{dt}=\frac{dI}{dt}$$

Analysis of the data in Table 1 shows that when the transition resistance value is lower than 800 Ω, all three time-domain characteristic quantities can achieve 100% fault phase selection accuracy. However, as the transition resistance increases to 1000 Ω, random noise interferes with the transient mutation signal of the surface magnetic field of the three-core cable, resulting in varying degrees of decline in the phase selection accuracy of each time-domain characteristic quantity. By calculating the difference between the maximum and minimum values of the signal, the difference value effectively amplifies the weak transient changes at the moment of the fault; therefore, it still maintains high phase selection reliability during high-resistance grounding, and the phase selection accuracy is better than the maximum and minimum values or the rate-of-change.

**Figure 5:**
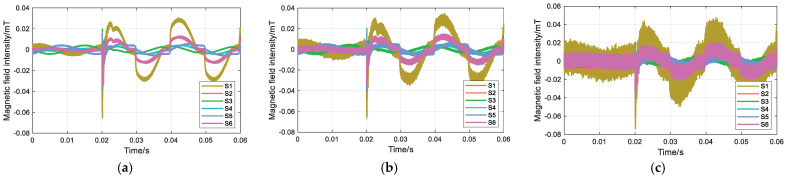
Influence of different SNRs: (**a**) 30 dB; (**b**) 20 dB; and (**c**) 10 dB.

In the simulation, noise with SNRs of 30 dB, 20 dB, and 10 dB is introduced to evaluate the influence of different noise levels on the proposed magnetic field intensity difference feature. A single-phase grounding fault on phase A is assumed, with the transition resistance set to 500 Ω. The rotation angles *α*, *β*, and *γ* are set to 3π/2, 5π/6, and π/6, respectively. The surface magnetic field waveforms under the three different SNR conditions are shown in Figure 5, and the corresponding magnetic field intensity differences at each measurement point are summarized in Table 2.

As shown in Table 2, under SNRs of 30 dB, 20 dB, and 10 dB, the magnetic field intensity difference at measurement point S1 is consistently the largest, and the faulted phase is correctly identified as phase A. These results indicate that the proposed time-domain feature is applicable in harsh noise environments. Moreover, the faulted phase can be accurately identified for single-phase grounding faults with transition resistance values below 800 Ω.

##### Spatial Characteristic Quantities

**Figure 6:**
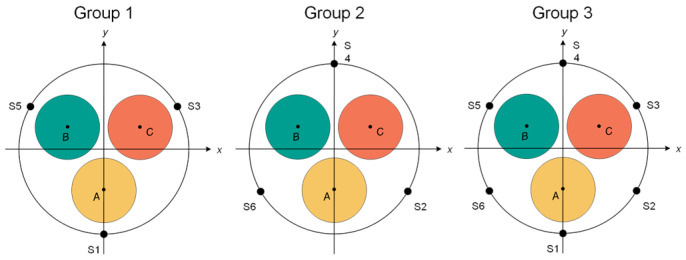
Working Condition 1.

**Figure 7:**
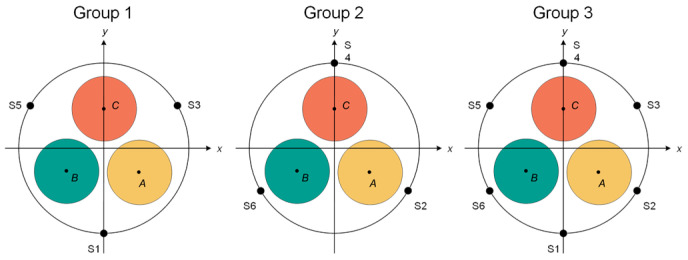
Working Condition 2.

To find the most suitable number of measurement points and combined spatial characteristic quantities on the surface of the annular TMR array, the six measurement points are divided into three groups: Group 1 is S1, S3, and S5; Group 2 is S2, S4, and S6; and Group 3 is S1, S2, S3, S4, S5, and S6. A metallic single-phase grounding fault occurs in phase A, and the magnetic field intensity differences in each measurement point in the three groups of measurement point combinations are detected. Working Condition 1: the rotation angles *α*, *β*, and *γ* are 3π/2, 5π/6, and π/6, respectively, as shown in Figure 6. Working Condition 2: the rotation angles *α*, *β*, *and γ* are 11π/6, 7π/6, and π/2, respectively, as shown in Figure 7.

Analysis of the data in Table 3 shows that under the above two specific angle conditions, in Group 2, S2, S4, and S6 measurement points of Working Condition 1, *B*_S2_^pp^ is equal to *B*_S6_^pp^, and both *B*_S2_^pp^ and *B*_S6_^pp^ are greater than *B*_S4_^pp^, making it impossible to select the phase correctly. In Group 1, S1, S3, and S5 measurement points of Working Condition 2, *B*_S1_^pp^ is equal to *B*_S3_^pp^, and both *B*_S1_^pp^ and *B*_S3_^pp^ are greater than *B*_S5_^pp^, which also makes it impossible to select the phase correctly. The third group of six measurement points, S1~S6, can all identify the fault phase. The number of different measurement points has an impact on the phase selection method proposed in this paper, and arranging six measurement points can realize effective fault phase selection of three-core cables.

#### 2.2.3. Faulted Phase Selection Process

**Figure 8:**
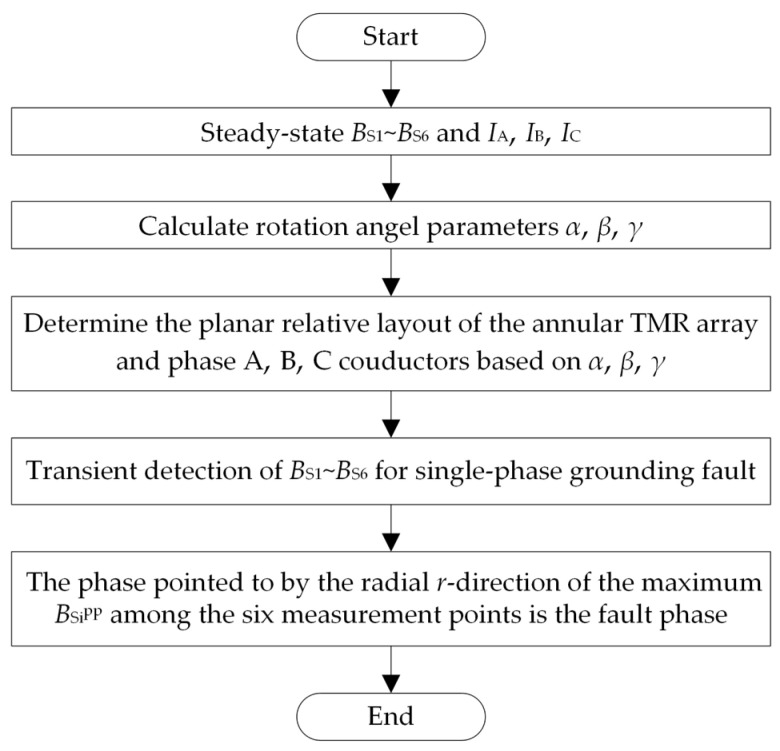
Procedure.

By combining the above steady-state inversion of the rotation angle and transient identification of fault phase for a single-phase grounding fault, the fault phase selection process is summarized in Figure 8.

## 3. Results

### 3.1. Simulation

**Figure 9:**
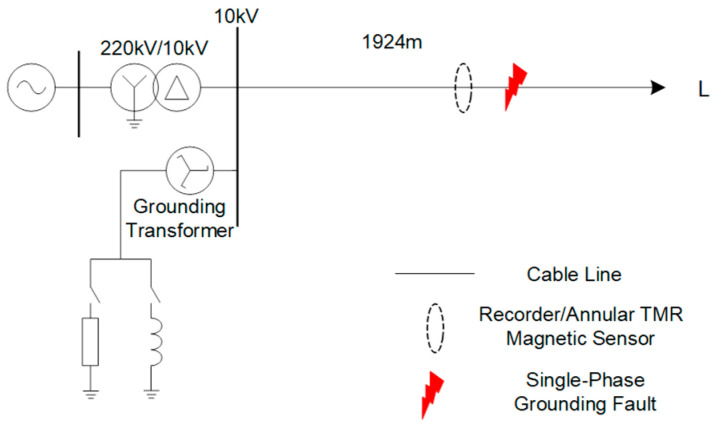
Single-phase grounding fault model.

To verify the proposed fault phase selection method for three-core cables, a medium-voltage cable feeder single-phase grounding fault simulation model is built in MATLAB/Simulink (R2023a). As shown in Figure 9, the transformer turns ratio is 220 kV/10 kV with a Y/△ connection, the arc suppression coil adopts an 8% over-compensation mode, and the small resistance is set to 10 Ω. A single-phase grounding fault is set in a 1924 m long cable feeder. The fault point is set 1301 m away from the 10 kV bus and 623 m away from the load. The detection point is arranged 200 m in front of the fault point. The line terminal is unloaded, and the sampling rate is 1 MHz.

The structural parameters of the three-core cable are shown in Table 4. The rotation angles *α*, *β*, and *γ* between OA, OB, OC, and the positive direction of the *x*-axis are 2π/3 apart. The cable radius *R* is 46.3 mm, and *r*_A_ = *r*_B_ = *r*_C_ = 21 mm.

Based on the three-phase currents obtained from the simulation, this paper substitutes them into the surface magnetic field formulas of *B*_S1_~*B*_S6_ to forward calculate the magnetic field intensity of the six measurement points S1~S6 on the surface of the three-core cable.

#### 3.1.1. Steady-State Inversion of Rotation Angle

Set *α*, *β*, and *γ* to 3π/2, 5π/6, and π/6, respectively. Based on the differential evolution algorithm, the three-phase currents *I*_A_, *I*_B_, and *I*_C_ and the surface magnetic field intensities *B*_S1_~*B*_S6_ obtained from the simulation at any time are inverted, and the obtained *α*, *β*, and *γ* parameters are shown in Table 5.

It can be seen from Table 4 that the minimum absolute error of the inverted rotation angle is 0.00111π, the maximum absolute error is 0.00311π, and the relative errors of *α*, *β*, and *γ* inversions are all less than 1%.

#### 3.1.2. Transient Phase Selection for Single-Phase Grounding Fault

##### Analysis of Fault Phase Selection for Three-Core Cables

**Figure 10:**
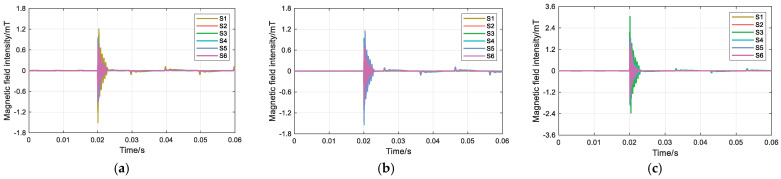
Magnetic field intensity at six measuring points during a single-phase grounding fault: (**a**) phase A grounding fault; (**b**) phase B grounding fault; (**c**) phase C grounding fault.

**Figure 11:**
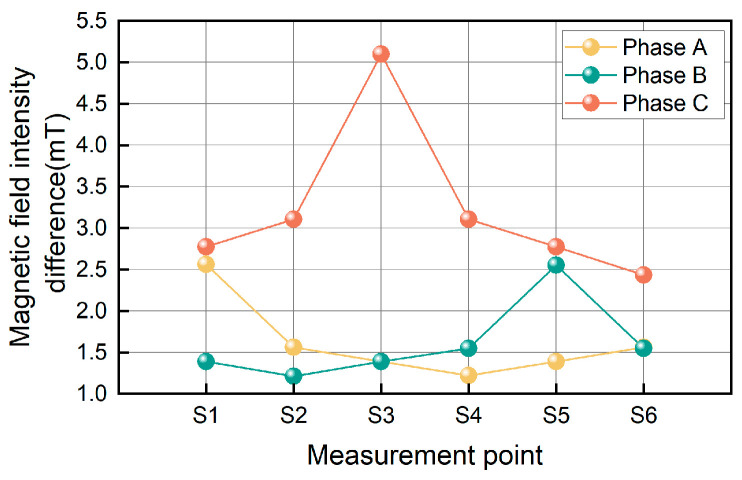
Magnetic field intensity difference at six measurement points for a single-phase grounding fault.

Taking an ungrounded neutral system as an example, the transition resistance at the fault point is set to 0 Ω, the rotation angles *α*, *β*, and *γ* are set to 3π/2, 5π/6, and π/6, respectively, and single-phase grounding faults occur in phases A, B, and C. The magnetic field intensity waveforms at S1~S6 are shown in Figure 10. Calculate the magnetic field intensity differences at the six measurement points S1~S6 on the surface of the three-core cable when single-phase grounding faults occur in phases A, B, and C, and obtain the magnetic field intensity difference curves as shown in Figure 11.

Based on the rotation angles *α*, *β*, and *γ* and the planar relative arrangement of the six measurement points of the annular TMR array, it can be concluded that when the magnetic field intensity difference *B*_S1_^pp^ of the S1 measurement point is the largest, a single-phase grounding fault occurs in phase A; when the magnetic field intensity difference *B*_S5_^pp^ at the S5 measurement point is the largest, a single-phase grounding fault occurs in phase B; when the magnetic field intensity difference *B*_S3_^pp^ at the S3 measurement point is the largest, a single-phase grounding fault occurs in phase C.

##### Influence of Different Rotation Angles

**Figure 12:**
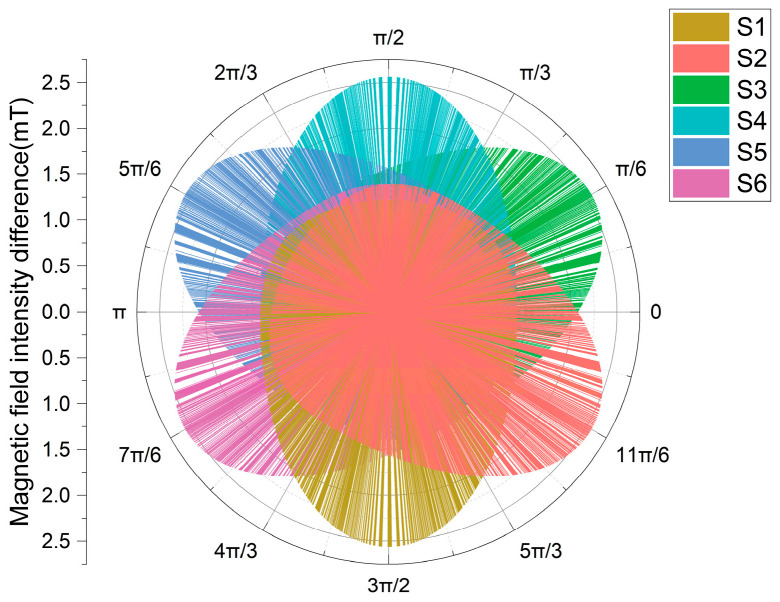
Magnetic field intensity differences at six measurement points under different rotation angles.

Different rotation angles affect the magnetic field intensity differences at the six measurement points. To eliminate situations where specific rotation angles result in two or more measurement points to obtain the same maximum magnetic field intensity difference, leading to phase selection errors. It is assumed that a single-phase grounding fault occurs in phase A. The rotation angle *α* is increased from 0 to 2π with an increment of π/1800 each time, and the resulting magnetic field intensity difference intervals at the six measurement points are shown in Figure 12.

Analysis shows that during the continuous change in the rotation angle *α* ∈ [0, 2π], the magnetic field intensity differences *B*_Si_^pp^ at measurement points S1~S6 show periodic distribution characteristics. For any measurement point Si, there exists an interval [*θ*_i1_, *θ*_i2_] ∈ [0, 2π] such that the magnetic field intensity difference in the measurement point within this interval satisfies the following:

(18) $$B_{{Si}^{pp}}(\alpha )>B_{{Sj}^{pp}}(\alpha )$$

In the formula, i ≠ j, i = 1, 2, …, 6; ∀*α* ∈ [*θ*_i1_, *θ*_i2_].

When *α* ∈ [1.334π, 5π/3], the magnetic field intensity difference *B*_S1_^pp^ of S1 is greater than that of other measurement points, and *B*_S1_^pp^ reaches its maximum when *α* = 3π/2. When *α* ∈ [1.668π, 0], *B*_S2_^pp^ is greater than that of other measurement points, and *B*_S2_^pp^ reaches its maximum when *α* = 11π/6. When *α* ∈ [π/1800, π/3], *B*_S3_^pp^ is greater than that of other measurement points, and *B*_S3_^pp^ reaches its maximum when *α* = π/6. When *α* ∈ [0.334π, 2π/3], *B*_S4_^pp^ is greater than that of other measurement points, and *B*_S4_^pp^ reaches its maximum when *α* = π/2. When *α* ∈ [0.667π, π], *B*_S5_^pp^ is greater than that of other measurement points, and *B*_S5_^pp^ reaches its maximum when *α* = 5π/6. When *α* ∈ [1.001π, 4π/3], *B*_S6_^pp^ is greater than that of other measurement points, and *B*_S6_^pp^ reaches its maximum when *α* = 7π/6.

Through the analysis of arbitrary rotation angles, it can be seen that different rotation angles have no effect on the phase selection method proposed in this paper.

##### Influence of Cable Core Eccentricity

From the above analysis, it can be concluded that when a single-phase grounding fault occurs in phase A and *α* = 3π/2, such that S1 is directly facing the phase A conductor and the geometric structure of the cable cores is symmetric, the magnetic field intensity difference at the S1 measurement point, *B*_S1_^pp^, should be greater than those at the other five measurement points. In practical operation, external mechanical compression of the conductors or cable eccentricity may occur, resulting in geometric asymmetry of the cable structure. Considering the eccentricity of the phase A conductor, the distance *r*_A_ from point *O* to the center of the phase A core, as well as the rotation angle α between *OA* and the positive *x*-axis, may vary within a certain range.

To verify the effectiveness of the fault phase selection method under cable eccentricity conditions, the parameter ranges are determined based on field operating experience. Specifically, the range of *r*_A_ is set to 19 mm~23 mm, and the range of the rotation angle α is set to 1.444π~1.556π. The distance from point *O* to the center of the phase B conductor is set to *r*_B_ = 20mm, with the rotation angle *β* between *OB* and the positive *x*-axis being 5π/6. The distance from point *O* to the center of the phase C conductor is set to *r*_C_ = 22 mm, with the rotation angle *γ* between *OC* and the positive *x*-axis being π/6. The cable radius *R* is 46.3 mm.

**Figure 13:**
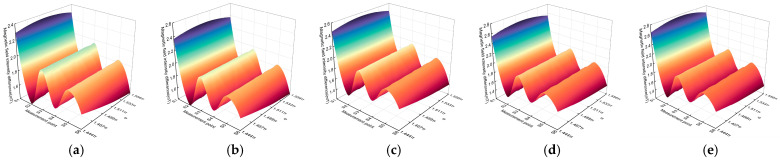
Simulation results of cable core eccentricity, (**a**) *r*_A_ = 19 mm; (**b**) *r*_A_ = 20 mm; (**c**) *r*_A_ = 21 mm; (**d**) *r*_A_ = 22 mm; (**e**) *r*_A_ = 23 mm.

This section investigates the variations in magnetic field intensity differences at different measurement points when the distance *r*_A_ and the rotation angle *α* deviate within the parameter ranges defined in the model. In the simulation, a single-phase grounding fault is assumed to occur on phase A. The variations in magnetic field intensity differences at different measurement points are shown in Figure 13. When *r*_A_ is set to 19 mm, 20 mm, 21 mm, 22 mm, and 23 mm, respectively, as the rotation angle *α* increases from 1.444π to 1.556π, the magnetic field intensity difference at the S1 measurement point, *B*_S1_^pp^, consistently remains higher than that at the other five measurement points. Within the range of α = 1.444π~1.556π, *B*_S1_^pp^ exhibits a symmetric distribution and reaches its maximum at *α* = 3π/2, corresponding to the condition where S1 is directly facing the phase A conductor.

##### Influence of Different Neutral Grounding Modes and Transition Resistances

There are three types of small current grounding systems in distribution networks: ungrounded neutral, neutral grounded through an arc suppression coil, and neutral grounded through small resistance. The amplitude of the fault phase current varies greatly among the three different grounding modes in the fault steady-state, and the change in the fault phase current further affects the change in the surface magnetic field intensity of the three-core cable. In the transient stage of a single-phase grounding fault, under the three different grounding modes, the fault phase current will experience a transient impact to increase, causing an increase in the surface magnetic field intensity of the three-core cable. The transition resistance range is set to 50 Ω~300 Ω, a single-phase grounding fault occurs in phase A, and the rotation angles *α*, *β*, and *γ* are 3π/2, 5π/6, and π/6, respectively.

**Figure 14:**
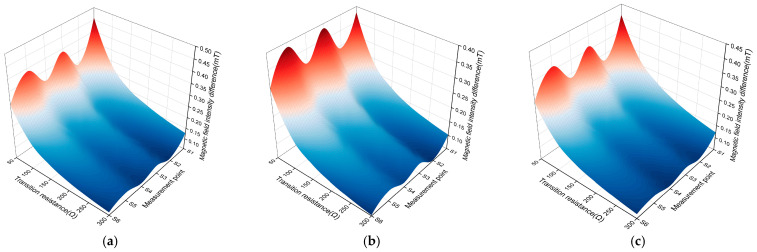
Simulation results of different neutral points and transition resistances: (**a**) ungrounded neutral; (**b**) arc suppression coil grounding; (**c**) low-resistance grounding.

The influence trend of the neutral point transition impedance on the magnetic field intensity difference is shown in Figure 14. Under the three neutral point grounding modes, the magnetic field intensity difference at the S1 measurement point is the largest, which is identified as phase A short circuit. Under the condition of non-high-resistance grounding at the fault point, different neutral point grounding modes and the value of the transition resistance have no effect on the phase selection method proposed in this paper.

### 3.2. Test Verification and Analysis

To further verify the effectiveness of the proposed method for fault phase selection in the case of single-phase grounding faults of three-core cables, a single-phase grounding fault is simulated on a 10 kV cable prototype test platform. The test site adopts two neutral point grounding modes: ungrounded and grounded through an arc suppression coil. The line length, fault point location, surface magnetic field measurement point layout, and load conditions are consistent with the simulation settings. The cable network frame is shown in Figure 8, and the structural parameters of the three-core cable are shown in Table 3. A single-phase grounding fault is set to occur in phase B during the test.

**Figure 15:**
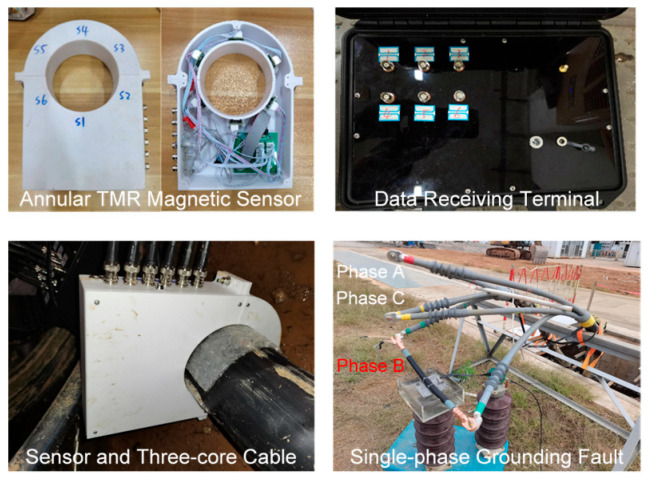
Test setup.

The test equipment and on-site layout are shown in Figure 15, including an annular TMR magnetic sensor composed of six TMR215x tunneling magnetoresistors and a six-channel acquisition card with a sampling rate of 1 MHz. A data receiving terminal with a built-in FPGA system board and an annular TMR magnetic sensor are arranged in the cable channel. The fault point uses a three-core cable with three-phase stripping. One phase conductor is drilled to the copper core, and the drilled conductor is immersed in water to simulate the actual cable immersion environment. The circuit breaker in the ring main unit is closed, and the system directly enters the single-phase grounding fault transient operation state. The annular Hall magnetic sensor is used for steady-state measurements in Reference [14] and is not applicable to single-phase grounding transient conditions.

The test is divided into two stages: system steady-state operation and single-phase grounding fault. In the early stage, the annular TMR magnetic sensor needs to be pre-installed on the surface of the three-core cable. In the steady-state operation stage, the system adopts the ungrounded neutral mode. The cable surface magnetic field and three-phase current data are collected through the annular TMR magnetic sensor and the oscilloscope, the rotation angles are calculated, and the planar relative arrangement of the three-phase conductors and the annular TMR array is determined. In the fault stage, two grounding modes are adopted: ungrounded neutral and grounded through an arc suppression coil. The surface magnetic field intensity of the three-core cable when a single-phase grounding fault occurs is recorded, and the fault phase selection is realized by calculating the magnetic field intensity difference during the transient process.

**Figure 16:**
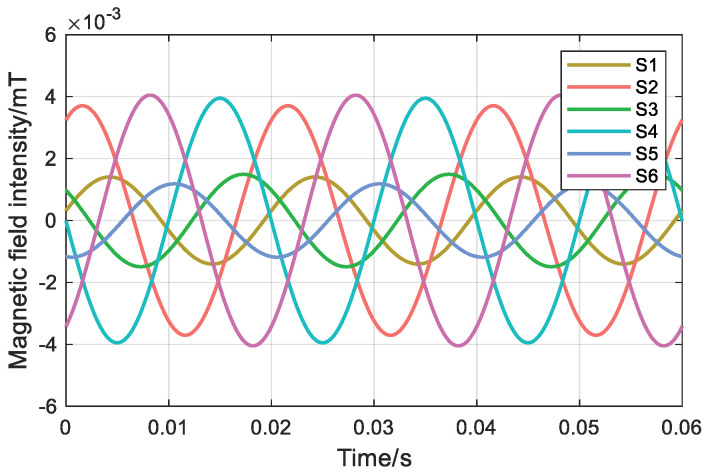
Magnetic field intensity at six measuring points.

During steady-state operation, the cable surface magnetic field is detected by the annular TMR magnetic sensor, and then the collected six magnetic field signals are denoised based on the wavelet transform to obtain the magnetic field waveforms of the six measurement points, as shown in Figure 16. The oscilloscope detects that the amplitudes of the A-, B-, and C-phase currents are 0.87 A.

Based on the measured magnetic field waveforms of the six measurement points and the current recordings, the three-phase currents *I*_A_, *I*_B_, and *I*_C_ and the surface magnetic field intensity *B*_S1_~*B*_S6_ at any time are inverted, and the calculated *α*, *β*, and *γ* parameters are 1.792π, 1.145π, and 0.411π, respectively. In actual operation, the three-core cable will be squeezed by external forces, resulting in eccentricity of the three-phase conductors inside the cable and making the rotation angles *α*, *β*, and *γ* between the three phases unable to be exactly 2π/3 apart.

**Figure 17:**
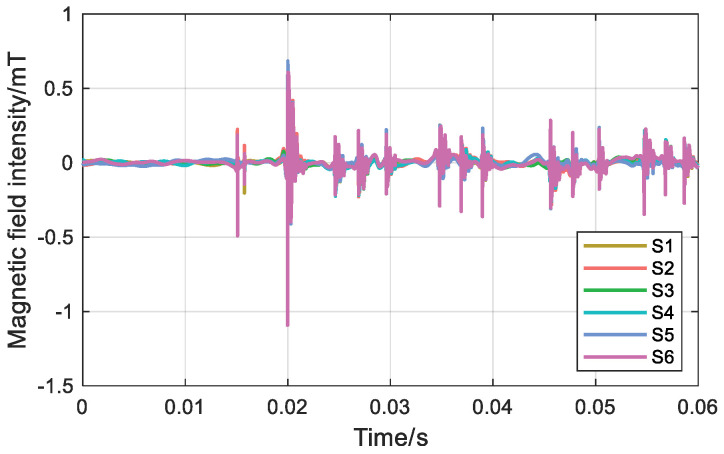
Magnetic field intensity at six points in an ungrounded neutral system.

**Figure 18:**
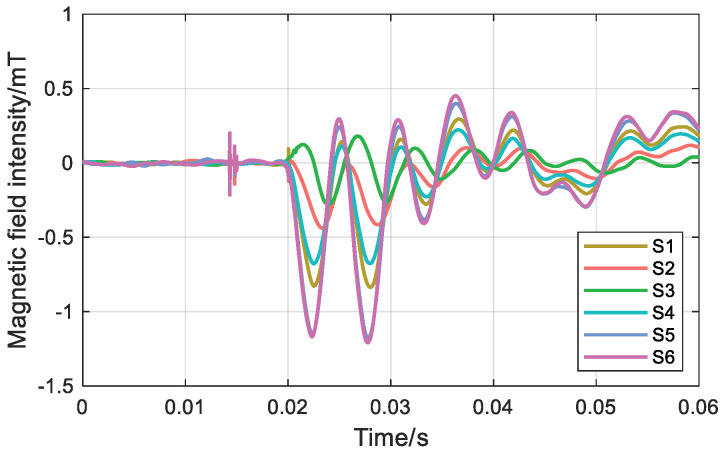
Magnetic field intensity at six points in a system with a neutral point grounded through an arc suppression coil.

During transient operation, the transient magnetic field waveforms of the S1~S6 measurement points are shown in Figure 17 and Figure 18. The spike at 0.015 s is an overvoltage caused by the closing of the circuit breaker and is not considered.

During the transient state of a single-phase grounding fault, for the ungrounded neutral mode, the magnetic field intensity differences at measurement points S1~S6 are 1.28 mT, 1.14 mT, 0.96 mT, 1.04 mT, 1.39 mT, and 1.75 mT, in sequence. For the neutral mode grounded via an arc suppression coil, the magnetic field intensity differences at measurement points S1~S6 are 1.16 mT, 0.56 mT, 0.46 mT, 0.90 mT, 1.58 mT, and 1.66 mT, in sequence. Under both grounding modes, the magnetic field intensity difference at measurement point S6 is the largest. 

Under steady-state conditions, the planar relative arrangement of the three-core cable and the annular TMR array is determined by the surface magnetic field and three-phase current. Under single-phase grounding fault transient conditions, analysis of the six measurement points shows that the magnetic field intensity difference at the S6 measurement point is the largest. Both different grounding modes identify the fault phase as phase B, which is consistent with the test fault phase setting.

The detection performance of the proposed method was quantitatively evaluated through repeated trials, with a total of 16 single-phase grounding fault tests conducted. Out of these, 15 trials successfully identified the faulted phase, yielding a TPR of 93.75%. Only one phase selection failure occurred, resulting in a false alarm rate of 6.25%, which was attributed to noise-induced triggering under low effective SNR conditions and a relatively low triggering threshold setting. The faulted phase was identified based on the magnetic field intensity difference feature following the occurrence of a single-phase grounding fault. A 20 ms data window was used to calculate the magnetic field intensity difference and complete faulted phase identification, ensuring that the detection latency does not exceed one fundamental power–frequency cycle. Additionally, assuming a binomial distribution of the detection results, the 95% confidence interval of the detection accuracy is approximately [70%, 99%]. The width of this confidence interval is primarily influenced by the limited number of experimental samples. Overall, despite the limited sample size, the proposed method shows high faulted phase selection reliability and strong potential for engineering applications under single-phase grounding fault conditions. As the device is deployed and more data is gathered, the statistical results are expected to become more stable and accurate.

## 4. Discussions and Limitations

This paper proposes a non-intrusive online fault phase selection method for 10 kV three-core cables based on an annular TMR magnetic sensor. The method combines steady-state sensing model parameter inversion and transient phase selection for single-phase grounding, which effectively solves the problem of fault phase identification in single-phase grounding faults. Compared with traditional fault phase selection methods, this method avoids invasive operations at both ends of the cable, reducing maintenance workload and shortening the fault handling period.

In terms of the selection of characteristic quantities, the proposed magnetic field intensity difference, as a time-domain characteristic quantity, can effectively amplify weak transient changes at the moment of the fault and maintain high phase selection reliability under high-resistance grounding conditions, which is superior to traditional maximum and minimum values and rate-of-change characteristic quantities. In terms of spatial characteristic quantities, by comparing the phase selection effects of different numbers of measurement points, it is verified that arranging six measurement points can avoid situations where phase selection cannot be performed correctly due to equal magnetic field intensity differences at partial measurement points, thereby ensuring the reliability of fault phase selection.

The simulation analysis shows that the differential evolution algorithm used in this paper has high inversion accuracy for the rotation angle, with a relative error of less than 1%, which lays a solid foundation for accurate fault phase selection. At the same time, the method is not affected by factors such as rotation angle and neutral grounding mode, which enhances the adaptability of the method. The prototype test further verifies the effectiveness of the method. Under two different neutral point grounding modes, the fault phase can be accurately identified, which confirms the engineering application value of the method.

This study also has certain limitations. The research focuses on the fault phase selection of single-phase grounding faults of three-core cables under non-high-resistance grounding conditions, and the applicability under high-resistance grounding conditions needs to be further studied. In addition, the influence of factors such as cable aging and temperature drift on the measurement accuracy of the sensor array has not been considered, which may affect the long-term stability of the method. Future research can focus on optimizing the algorithm to improve the phase selection accuracy under high-resistance grounding conditions and carry out research on temperature compensation and aging adaptation of the sensor array to further enhance the engineering application prospects of the method.

Temperature variation and long-term drift are inherent characteristics of magnetoresistive sensors, including TMR devices, and may affect absolute magnetic field measurements. However, the proposed faulted phase selection method does not rely on absolute magnetic field amplitudes. Instead, it is based on a magnetic field intensity difference feature extracted within a short fault transient window. This differential feature effectively suppresses slow-varying offset drift and low-frequency temperature-induced variations, which typically evolve over time scales much longer than the fault transient duration. Under practical operating conditions, temperature changes within the short fault transient interval are negligible compared with long-term environmental variations. Moreover, the proposed phase selection criterion depends on the relative magnitude relationship of *B*_Si_^pp^ among multiple measurement points rather than on their absolute values. Since all TMR sensors are mounted on the same annular structure and operate under nearly identical thermal conditions, temperature-induced drift predominantly manifests as a common-mode component, thereby exerting limited influence on the relative ranking used for faulted phase identification. From an implementation perspective, mature temperature compensation techniques can be readily applied, including periodic offset calibration under steady-state conditions, reference-based compensation using integrated temperature sensors, and software-based baseline tracking. These methods are well established in industrial TMR sensing systems and introduce minimal computational or hardware overhead.

Regarding calibration frequency, sensor offset and rotation angle parameters can be considered quasi-static under normal operating conditions. Consequently, calibration is generally required only during initial installation or commissioning, after sensor repositioning, or following prolonged operation or significant environmental changes, rather than on a frequent basis.

In the prototype test, the single-phase grounding fault is introduced by breaker closing. While this method allows for controllable and repeatable fault initiation, it may also introduce operation-induced transient disturbances. In contrast, practical single-phase grounding faults are primarily caused by sudden insulation breakdown, which occurs randomly and typically results in a steeper current rise and richer high-frequency transient components. These differences mainly affect the detailed transient waveform characteristics in the time-domain. However, the proposed phase selection method does not depend on specific transient waveform shapes or frequency-domain features. Instead, it is based on the spatial distribution of the surface magnetic field intensity differences around the three-core cable. Regardless of whether the fault is triggered by breaker closing or sudden insulation breakdown, a single-phase grounding fault causes the abrupt appearance of zero-sequence current and a dominant increase in current in the faulted phase. As a result, the measurement point closest to the faulted phase consistently shows the maximum magnetic field intensity differences, which form the foundation of the proposed phase selection criterion. Therefore, although the two fault scenarios differ in transient signal details, they are equivalent in terms of the spatial magnetic field variation characteristics used for phase selection, and the proposed method remains effective.

In practical field environments, strong electromagnetic pulse interference may introduce short-duration spikes in the measured magnetic field signals. The proposed method mitigates the impact of such interference through the following mechanisms. First, *B*_Si_^pp^ is extracted over a predefined transient time window, reducing the likelihood that pulse interference dominates the extrema. Second, the phase selection criterion is based on the relative magnitude of *B*_Si_^pp^ among multiple spatially distributed measurement points. Global pulse interference will affect all sensors similarly, without changing the relative ranking, while localized interference is typically short-lived and unlikely to dominate the maximum–minimum difference. Additionally, preprocessing techniques such as band-limited filtering, outlier rejection, or median smoothing can further suppress impulsive noise without affecting fault-induced magnetic field variation. Although strong pulse interference may introduce transient disturbances, it is unlikely to cause incorrect phase selection, and the proposed method demonstrates robust performance in practical field environments.

## 5. Conclusions

Based on the annular TMR magnetic sensor, this paper constructs a two-step non-intrusive online fault phase selection method for 10 kV belted three-core cables. The method integrates a steady-state sensing model parameter inversion and transient phase selection under a single-phase grounding fault. The effectiveness of the proposed phase selection method is verified through simulation analysis and experiment. The main conclusions are as follows:

(1) Under steady-state conditions, the differential evolution algorithm is used to invert the rotation angles *α*, *β*, *and γ* of the sensing model, thereby determining the planar relative arrangement of the sensor array and the three-core cable. Through simulation calculations, the relative error of the rotation angle inversion is less than 1%, which verifies the accuracy of the inversion algorithm.
(2) Under transient conditions of a single-phase grounding fault, based on the annular TMR array, a magnetic field intensity difference characteristic quantity is proposed. Through the maximum magnetic field intensity difference among the six measurement points in the annular TMR array, combined with the planar relative arrangement of the sensor array and the three-core cable, identification of the fault phase is realized. Moreover, the method is not affected by factors such as rotation angle and neutral grounding mode.

The annular TMR array can be used in the field of medium-voltage distribution network fault diagnosis. Future work will continue to expand the application scope of sensors in cable networks and carry out research on fault line selection and fault location based on the surface magnetic field of three-core cables.

## Figures and Tables

**Table 1:** Phase selection accuracy rate of each time-domain characteristic quantity.

Transition Resistance	Difference Accuracy	Maximum/Minimum Accuracy	Rate-of-Change Accuracy
0 Ω~800 Ω	100.0%	100.0%	100.0%
800 Ω~1000 Ω	99.0%	96.5%	94.5%

**Table 2:** Differences between measurement points under different SNRs.

SNR	S1	S2	S3	S4	S5	S6
30 dB	0.096 mT	0.045 mT	0.055 mT	0.042 mT	0.055 mT	0.046 mT
20 dB	0.101 mT	0.047 mT	0.056 mT	0.042 mT	0.056 mT	0.049 mT
10 dB	0.121 mT	0.058 mT	0.057 mT	0.045 mT	0.058 mT	0.060 mT

**Table 3:** Differences between measurement points.

Working Condition	S1	S2	S3	S4	S5	S6
1	2.54 mT	/	1.39 mT	/	1.39 mT	/
	/	1.56 mT	/	1.22 mT	/	1.56 mT
	2.56 mT	1.56 mT	1.39 mT	1.22 mT	1.39 mT	1.56 mT
2	1.56 mT	/	1.56 mT	/	1.22 mT	/
	/	2.56 mT	/	1.39 mT	/	1.39 mT
	1.56 mT	2.56 mT	1.56 mT	1.39 mT	1.22 mT	1.39 mT

**Table 4:** Structural parameters of the three-core cable.

Structure	Radius/mm	Material
Conductor	11.6	Copper
Inner Semiconductive Layer	12.4	Semiconductive Polymer
XLPE Insulation Layer	17.1	XLPE
Outer Semiconductive Layer	17.7	Semiconductive Polymer
Copper Tape Shield Layer	17.8	Copper
Steel Tape Shield Layer	41.6	Galvanized Steel
Outer Sheath Layer	46.3	PVC

**Table 5:** Rotational angle inversion.

Rotation Angle	Actual Value	Inverted Value	Absolute Error	Relative Error
*α*	3π/2	1.504π	0.00311π	0.21%
*β*	5π/6	0.831π	0.00206π	0.25%
*γ*	π/6	0.166π	0.00111π	0.67%

## Data Availability

The data used to support the findings of this study are available from the corresponding author upon reasonable request.

## References

[1] Li M., Zhou C., Zhou W., Wang C., Yao L., Su M., Huang X. (2018). A Novel Fault Location Method for a Cross-Bonded HV Cable System Based on Sheath Current Monitoring. Sensors.

[2] Li M., Liu J., Zhu T., Zhou W., Zhou C. (2019). A Novel Traveling-Wave-Based Method Improved by Unsupervised Learning for Fault Location of Power Cables via Sheath Current Monitoring. Sensors.

[3] Emdadi K., Gandomkar M., Aranizadeh A., Vahidi B., Mirmozaffari M. (2025). Overview of Monitoring, Diagnostics, Aging Analysis, and Maintenance Strategies in High-Voltage AC/DC XLPE Cable Systems. Sensors.

[4] Li Y., Xu J., Wang P., Li G. (2025). Research on Arc Extinguishing Characteristics of Single-Phase Grounding Fault in Distribution Network. Energies.

[5] Deng F., Zeng X., Tang X., Li Z., Zu Y., Mei L. (2020). Travelling-wave-based fault location algorithm for hybrid transmission lines using three-dimensional absolute grey incidence degree. Int. J. Electr. Power Energy Syst..

[6] Kulikov A., Loskutov A., Ilyushin P., Kurkin A., Sluzova A. (2025). High-Voltage Overhead Power Line Fault Location Through Sequential Determination of Faulted Section. Technologies.

[7] Zhang W., Song Y., Wu X., Liu H., Tian H., Tang Z., Xu S., Chen W. (2025). Detecting Partial Discharge in Cable Joints Based on Implanting Optical Fiber Using MZ–Sagnac Interferometry. Sensors.

[8] Yang Z., Song J., Li Y., Zhang Y. Analysis of HVDC Commutation Failure Influence on AC Phase Selector Based on Fault Component. Proceedings of the 2019 IEEE 8th International Conference on Advanced Power System Automation and Protection (APAP).

[9] Li Y., Xu G., Li W., Lu Z., Wang C. (2020). Influence of the integration of MMC-HVDC station on traditional phase selection method and the countermeasure. Trans. Chin. Electrotech. Soc..

[10] Lin S., He Z., Zang T., Qian Q. (2010). Novel approach of fault type classification in transmission lines based on rough membership neural networks. Proc. CSEE.

[11] He Z., Fu L., Lin S., Bo Z. (2010). Fault detection and classification in EHV transmission line based on wavelet singular entropy. IEEE Trans. Power Deliv..

[12] Dash P.K., Das S., Moirangthem J. (2015). Distance protection of shunt compensated transmission line using a sparse S-transform. IET Gener. Transm. Distrib..

[13] Wei D., Gong Q., Lai W., Wang B., Liu D., Qiao H. (2016). Distance protection of shunt compensated transmission line using a sparse S-transform. Proc. CSEE.

[14] Wang H., Huang B., Liao Y.Q. (2025). Phase current measurement of three-core cables based on inverse analysis of surface magnetic field. Proc. CSEE.

[15] Zhao Z., Xiao J., Li H., Wang H. (2025). Surface Magnetic Field and Phase Current Sensing of Steel Tape-Wrapped 3-Core MV Cables. Sensors.

[16] Zhou G., Xiao C., Liu D. (2012). Remanent magnetic field modeling of naval vessels based on integral method and Tikhonov regularization method. Acta Armamentarii.

[17] Sun X., Lee W.K., Hou Y., Pong P.W. (2014). Underground Power Cable Detection and Inspection Technology Based on Magnetic Field Sensing at Ground Surface Level. IEEE Trans. Magn..

[18] Chan J.Y.C., Norman C.F., Lai L.L. (2013). A coreless electric current sensor with circular conductor positioning calibration. IEEE Trans. Instrum. Meas..

[19] Weiss R., Makuch R., Itzke A., Weigel R. (2017). Crosstalk in Circular Arrays of Magnetic Sensors for Current Measurement. IEEE Trans. Ind. Electron..

[20] Itzke A., Weiss R., DiLeo T., Weigel R. (2018). The Influence of Interference Sources on a Magnetic Field-Based Current Sensor for Multiconductor Measurement. IEEE Sens. J..

[21] Yu H., Qian Z., Liu H., Qu J. (2018). Circular array of magnetic sensors for current measurement: Analysis for error caused by position of conductor. Sensors.

[22] Di Rienzo L., Bazzocchi R., Manara A. (2001). Circular arrays of magnetic sensors for current measurement. IEEE Trans. Instrum. Meas..

[23] Zhu K., Han W., Lee W.K., Pong P.W. (2017). On-site non-invasive current monitoring of multi-core underground power cables with a magnetic-field sensing platform at a substation. IEEE Sens. J..

[24] Zhu K., Lee W.K., Pong P.W. (2017). Energization-status identification of three-phase three-core shielded distribution power cables based on non-destructive magnetic field sensing. IEEE Sens. J..

[25] Hu J., Ma H.Y., Li P. (2023). Research progress of current measurement method based on magnetic sensor array. High Volt. Eng..

[26] Ye N., Zhi C., Yu Y., Lin S., Liu F. (2025). TCN-LSTM-AM Short-Term Photovoltaic Power Forecasting Model Based on Improved Feature Selection and APO. Sensors.

[27] Zhou F., Wang W., Xiao Y., Zhou C. (2026). Simulated Annealing–Guided Geometric Descent-Optimized Frequency-Domain Compression-Based Acquisition Algorithm. Sensors.

[28] Zhang T., Peng X., Liu Y., Yin K., Li P. (2025). A Contrastive Representation Learning Method for Event Classification in Φ-OTDR Systems. Sensors.

[29] Zhou D., Xing F., Liu W., Liu F. (2025). Highly Accelerated Dual-Pose Medical Image Registration via Improved Differential Evolution. Sensors.

